# Controlled clinical trials in cancer pain. How controlled should they be? A qualitative systematic review

**DOI:** 10.1038/sj.bjc.6603162

**Published:** 2006-05-16

**Authors:** R F Bell, T Wisløff, C Eccleston, E Kalso

**Affiliations:** 1 Regional Centre of Excellence in Palliative Care Western Norway/Institute for Surgical Sciences/Pain Clinic Haukeland University Hospital, N-5021 Bergen, Norway; 2 Norwegian Knowledge Centre for the Health Services, Oslo, Norway; 3 Pain Management Unit, Royal National Hospital for Rheumatic Diseases/University of Bath, UK; 4 Pain Clinic, Helsinki University Central Hospital/University of Helsinki, Finland

**Keywords:** systematic review, methodology, opioids, cancer pain

## Abstract

This qualitative systematic review of the clinical methodology used in randomised, controlled trials of oral opioids (morphine, hydromorphone, oxycodone) for cancer pain underlines the difficulties of good pain research in palliative care. The current literature lacks placebo-controlled superiority trials. Recommendations for future research are discussed.

Conducting randomised controlled trials in the palliative-care patient population is a challenge. It is difficult to recruit patients and to conduct trials successfully due to the serious nature of the illness and the inevitability of symptom progression.

Pain trials are especially prone to error. Pain is a subjective experience and as such is influenced by a number of variables that are difficult to control, both in the clinical situation, and in the context of a controlled trial. Psychological factors such as anxiety and depression may influence the perception of pain and even the effect of opioids (Wasan *et al*, 2005). A critical review of the literature on cancer pain found strong evidence for a relationship between psychosocial factors and chronic cancer pain (Zaza and Baine, 2002). The authors concluded that cancer pain assessment should include routine screening for psychological distress. Cognitive style such as catastrophising may also contribute to the intensity of pain (Sullivan *et al*, 2001; Keefe *et al*, 2005). Depression, anxiety and sleep disturbance are common in the cancer patient population. It would therefore seem prudent to consider these variables when designing cancer pain trials.

The objective of this review was to conduct a systematic investigation of specific oral opioid (morphine, oxycodone, hydromorphone) pain trials in adult cancer patients in order to

1. evaluate the general methodological quality of randomised, controlled trials of opioids in cancer pain
2. identify factors related to poor methodological quality
3. investigate whether psychological factors are routinely addressed in opioid trials
4. make recommendations for future clinical research on pain treatment in palliative care

It was decided to restrict the review to oral opioids in order to have consistency and to minimise variation in the studies, concentrating on study drugs that behave in a similar manner.

## MATERIALS AND METHODS

### Search strategy and selection criteria

Search terms were oxycodone, morphine, hydromorphone, cancer, using the Boolean operators ‘OR’ and ‘AND’. The search was performed in the Cochrane Central Register of controlled trials (CENTRAL) (current issue), The Cochrane Database of Systematic Reviews (current issue), MEDLINE (1966 – January 2005) and EMBASE (1980 – January 2005). Abstracts and unpublished reports were not considered. There was no language restriction. The date of the most recent search (CENTRAL) was 9 November 2005.

All identified records from each of the databases were examined. Studies in adult patients 18 years and above involving treatment of chronic cancer pain with specific oral opioid (morphine, oxycodone or hydromorphone) were considered. The titles and abstracts of studies were examined independently by two reviewers (RFB, EK) and potentially relevant studies were retrieved for assessment for inclusion in the review. Each trial report that appeared to meet the criteria was independently assessed for inclusion by three reviewers (RB, CE, EK).

### Validity assessment

Study quality (randomisation/allocation concealment; details of blinding measures, withdrawals and dropouts; overall quality score) were evaluated using the three item (1–5) Oxford Quality scale (Jadad *et al*, 1996). Validity was evaluated using the five item (1–16) Oxford Pain Validity Scale (OPVS) (Smith *et al*, 2000). Scoring was performed independently by three reviewers (RFB, CE, EK). The statistical analyses employed in the individual trials used were assessed by a statistician (TW).

### Data abstraction

A data extraction form was designed and the following data items were collected:

1. Publication details,
2. Patient population, number of patients
3. Exclusion criteria
4. Description of pain
5. Psychological variables
6. Design, study duration and follow-up
7. Outcome measures
8. Withdrawals and adverse effects
9. Acknowledgement of pharmaceutical industry
10. Statistics

### Study characteristics

Randomised trials, described as double-blind and having either placebo or active controls were included.

### Quantitative data synthesis

This is a qualitative systematic review. Quantitative analysis was not performed.

Quality of Reporting of Meta-analyses (QUOROM) (Moher *et al*, 1999) guidelines were followed.

## RESULTS

### Study characteristics

Thirty-four randomised, double-blinded trials were identified. The characteristics of the included trials are summarised in Table 1. Seventeen trials were described as multicentre trials, or enrolled patients from more than one centre. In one trial, 85 patients were recruited from 30 general practice, hospital or hospice locations (O’Brien *et al*, 1997). A total of seven trials enrolled a hundred or more patients in each trial. Six of these were multicentre trials with the number of centres involved ranging from seven to 19. The maximum number of patients enrolled in any one trial was 180. This was a multicentre trial involving 17 centres (Kaplan *et al*, 1998). In general, the multicentre trials recruited larger numbers of patients than the single-centre trials. The mean number of patients enrolled in a multicentre trial was 80, more than twice that of the mean number in a single-centre trial.

### Patients

The total number of patients enrolled was 1864. Patients recently or currently receiving radiotherapy and/or chemotherapy were specifically excluded in 20 of 34 trials. In two of these trials (Kaplan *et al*, 1998; Parris *et al*, 1998), the protocol was subsequently changed to facilitate patient inclusion.

### Trial design

Twenty-six trials had crossover, and eight had parallel group design; 26 trials used double-dummy technique. Thirty-three of the 34 trials were equivalency studies.

Only one study (Hoskin *et al*, 1989) had a placebo control, while another study had a placebo arm in the first phase (Broomhead *et al*, 1997). Only nine studies described the process of randomisation.

### Quality, validity and sensitivity

Quality scores were generally high with a mean of 4, while validity scores were somewhat lower with a mean of 10 on the OPVS scale of 1–16.

Only nine trials were scored as sensitive. In the remaining trials, baseline levels of pain were insufficient to be able to measure a change following treatment, baseline levels of pain could not be assessed or internal sensitivity was not demonstrated.

### Group size

Six studies had a group size between 10 and 20, while 28 studies had a group size over 20.

### Duration

Ten trials had a duration of 7 days or less. Fourteen trials had a duration of between 7 and 14 days. Ten trials lasted longer than 2 weeks. The trial with the longest duration lasted maximum 35 days (Stambaugh *et al*, 2001).

### Withdrawals/dropouts

Twenty-nine studies had a withdrawal/dropout/nonevaluable rate over 10%, with 12 studies exceeding 30%. Six trials had a withdrawal rate of 40% or more, including one study with a maximum duration of 28 days and a withdrawal rate of 44% (Coluzzi *et al*, 2001). The most common reason for failure to complete the study was adverse effects, followed by insufficient pain relief and deterioration due to disease progression. In general, trials of longer duration had larger numbers of patients who failed to complete the study. Twenty-four studies had a duration of more than 1 week, with 16 studies lasting 2 weeks or longer.

### Pain description and assessment

Only 11 of 34 studies (Table 1, trials 5, 6, 8, 12, 15, 18, 19, 26, 28, 30, 34) included a description of the pain. In two of these (Deschamps *et al*, 1992; Stambaugh *et al*, 2001), the description was restricted to the location of the pain. Five trials (Hays *et al*, 1994; Bruera *et al*, 1996, 1998, 2004; Hagen and Babul, 1997) evaluated patients using the Edmonton staging system which classifies pain as visceral, bone, soft tissue, neuropathic, mixed, unknown and incidental or nonincidental. However, only three of these trials reported data on the type of pain.

Pain intensity was assessed in all trials: in nine trials using visual analogue scale (VAS), in seven using verbal rating scale (VRS) and in four trials using a numerical rating scale (NRS). Thirteen trials used VAS in addition to VRS. One trial used a nonvalid assessment, nurse-rated VRS that was later converted to a numerical score. Five trials rated pain relief in addition to pain intensity. In three of these trials, pain relief was assessed using VAS and in two trials using VRS.

The criteria for adequate/inadequate pain relief was clearly defined in only eight of the 34 trials. The criteria differed for each of these trials and for adequate pain relief included: ‘maximum 3 on a 7 point VRS, and not more than two daily requests for rescue analgesia’ (Klepstad *et al*, 2003); ‘no need for dose adjustment for three or more days and no morphine sulphate solution intake exceeding 50% of the daily morphine dosage supplied by the test drug’ (Deschamps *et al*, 1992); ‘no more than three supplementary doses of immediate release morphine per day’ (Portenoy *et al*, 1989); ‘required daily rescue doses over 2 days interval not more than 20% of the total daily morphine doses’ (Cundiff *et al*, 1989); ‘over a 48-h period, the q12h dose was unchanged, less than two supplemental analgesic doses were taken per day, the dosing regimen for any non-opioids or adjuvants was unchanged, and the patient reported that pain control was acceptable and any side effects were tolerable’ (Mucci-LoRusso *et al*, 1998). Inadequate pain relief was defined as: ‘more than two doses of rescue medication/24 h, or moderately severe global pain score’ (Stambaugh *et al*, 2001); ‘despite dose escalation, pain intensity rating more than three on a five point VRS’ (Wilder-Smith *et al*, 1994). One study defined a clinically meaningful difference in VAS scores as 25 mm on a 100 mm scale (Walsh *et al*, 1992).

### Psychological variables and sleep (see Table 1)

Despite the fact that anxiety and depression are known to influence the perception of pain, only three trials (Walsh, 1985; Walsh *et al*, 1992; Finn *et al*, 1993) assessed and reported these variables. In addition, one of these trials and two others assessed and reported ‘mood’ and a third trial used Brief Pain Inventory (BPI) ratings of mood and enjoyment of life. A further five trials used the Edmonton staging system that includes assessment of the degree of psychological stress in order to calculate a prognosis score, but did not report data on psychological variables. One of these trials in addition used the Folstein Mini-Mental status. One trial (Heiskanen and Kalso, 1997) used the Modified Specific Drug Effects Questionnaire (MSDEQ) which includes questions such as ‘Do you feel anxious?’ and ‘Do you feel relaxed ?’. Mucci-LoRusso *et al* (1998) used a quality of life questionnaire, the Functional Assessment of Cancer Therapy-General (FACT G), which includes an emotional subscale. Nine trials assessed sleep and seven of these provided data.

### Adverse effects

All trials included data on adverse events. Twenty-five trials assessed adverse event severity using verbal or categorical rating scales, or VAS. Eighteen trials provided data from these measurements.

Adverse effect intensity was rated using VAS in nine trials, NRS in one trial and by categorical or verbal rating scale in 13 trials. One trial used both VAS and categorical scales. In one trial, where severity was investigator-rated, it is unclear which method was used (Kaplan *et al*, 1998).

A total of 18 trials provided dichotomous data on the incidence of adverse effects.

These included all nine studies not grading the intensity of adverse effects. Only nine of the 25 studies grading adverse effect intensity provided dichotomous data on incidence.

### Statistical methods

The statistical methods used are summarised in Table 2. All 34 trials are judged to have chosen appropriate statistical methods on most of the analyses; however, some problems were identified regarding the statistical analysis in 18 trials.

In nine trials (Table 2, trials 1, 4, 10, 12, 15, 19, 20, 25, 33), the authors concluded that the test drug was equally effective as the comparator drug. However, the tests performed only show no evidence of effect, not evidence of no effect.

Only 10 of 34 trials reported to have performed *pre-hoc* sample-size calculation. In three trials, some posterior power calculations were performed (Portenoy *et al*, 1989; Walsh *et al*, 1992; Mignault *et al*, 1995) and in one study it is unclear whether sample-size calculation was performed (Stambaugh *et al*, 2001). In other words, more than 50% of the trials did not report performing power calculations.

### Sponsored research

The pharmaceutical industry was specifically acknowledged in 24 of 34 trials as follows: co-authors (18 trials) financial support (four trials), manufacture of placebo double-dummy medication (one trial) morphine assays (one trial). Twenty-three of these 24 trials were equivalency studies.

## DISCUSSION

This review has identified several factors/areas that could improve the methodological quality of studies on cancer pain. The findings of the review also suggest that specific validity scores should be developed to focus more on factors that are relevant in cancer pain.

### Factors influencing methodological quality

#### The research question

The most commonly asked research question in these trials is whether one opioid is as good as another, or whether two forms of administration of the same opioid are equally effective. However, what we really need to know is more about factors which influence the cancer patient's experience of pain, and which factors influence treatment outcome. In order to do this, we need to define good and bad responders and to identify factors that influence treatment outcome. It is important to understand why some patients do not achieve pain relief, for example with opioids, and why some patients respond to one opioid but not to another.

#### Trial design

Thirty-three of the 34 trials in this review are equivalency (or non-inferiority) studies comparing two opioids or two or more formulations of the same opioid.

Problems with equivalency trials: In equivalency studies of analgesics (drug A *vs* drug B), the focus is a comparison of the test drug with standard therapy (active control), not efficacy of the test drug *per se*. Equivalency trials are potentially problematic since they do not measure efficacy directly. In such a trial, the same result is consistent with three possible conclusions (Landow, 2000; Moore *et al*, 2003):

- Both treatments are equally effective
- Both treatments are equally ineffective
- The trials were inadequate to detect differences between treatments

This problem may be avoided if the control has previously in the same patient population been shown to be effective compared to placebo. This is not the case in cancer pain, as trials having a placebo control are lacking.

**Figure 1:**
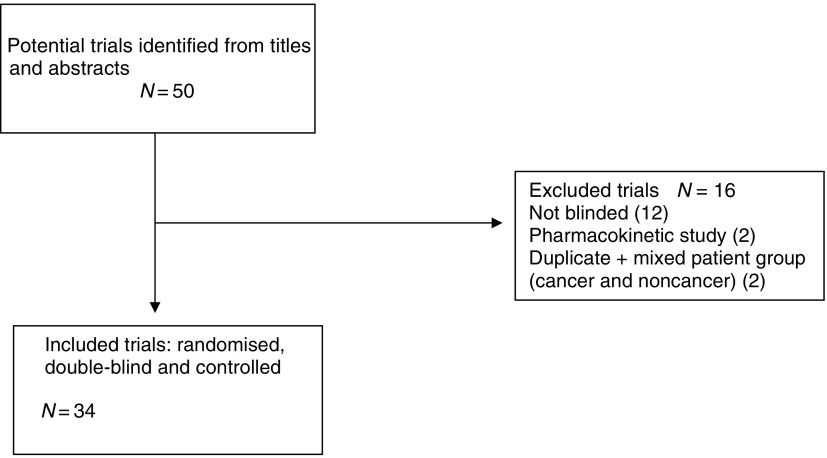
QUOROM statement flowchart.

Equivalency trials have important methodological limitations and must be rigorously performed if they are to produce reliable conclusions, for example needing substantially more patients than their placebo-controlled counterparts (Jones *et al*, 1996). The majority of trials in this review were underpowered (Figure 1).

Placebo control *vs* active comparator: Since patients in pain respond to placebo, we need placebo-controlled trials to reliably determine opioid efficacy. Many researchers consider that it is unethical to use a placebo control in trials of cancer pain. However, it is common to use placebo controls both in acute pain and in chronic pain trials. Morphine is accepted as the gold standard for cancer pain treatment, however high-quality placebo-controlled efficacy data in cancer pain is lacking. Extrapolation of efficacy data from trials in other patient populations is generally not advised.

Using a placebo-control where possible would also permit smaller group sizes. We do not suggest that a placebo control should be used in all cancer pain studies, but that it is feasible in certain types of trial. While it is not possible to randomise patients treated with stronger opioids to a placebo group, patients using weaker opioids may be randomised to a placebo group. Almost half of the studies included in this review recruited patients being treated with WHO step 2 (weaker) opioids. In these studies, it would have been possible to include a placebo-arm, provided the patients had free access to normal-release opioid as rescue medication, and using consumption of rescue medication as the primary outcome measure. This type of study should have a limited duration, for example 14 days, and should not present ethical problems since the treatment is similar to the clinical treatment of breakthrough pain, and would be expected to give satisfactory pain relief. Indeed, the ethics of using a placebo control in this kind of design should be compared to the potential ethical dilemma of exposing seriously ill patients to trials which do not produce reliable results due to lack of power, sensitivity or other methodological problems.

Crossover or parallel group?: A crossover design may be useful as it increases the power of the study and uses the patient as his/her own control. Crossover trials are important since they can identify clear patient preferences for one drug over another and suggest ideas for future research for the mechanisms behind these differences. Crossover trials should have as short a duration as possible in order to reduce number of withdrawals, while parallel group trials allow longer follow-up with regular assessment of outcomes.

### Reporting of data

Since trial size is a general problem, efforts should be made to enable combination of data from different trials (meta-analysis). Data should be given as means±s.d., or medians+range together with responder status. The latter will help those who perform meta-analysis and also enable the researchers to further analyse the reasons why some patients respond to analgesic drugs and others do not. Adverse effects should be reported as dichotomous data. Patient treatment preference is valuable information and should be recorded. For example, some adverse effects may be more acceptable than others.

### Pain description and assessment

Cancer pain may be constant, intermittent or both. It may be nociceptive, neuropathic or mixed. It may be cancer-related or treatment-related. If we are to investigate opioid efficacy, we at least need to know what kind of pain is being treated. In a parallel group study, if there are more patients in one group having neuropathic pain, then this would be expected to influence opioid treatment outcome.

As a minimum requirement, each patient included in a pain trial should be assessed specifically for pain and given a simple pain diagnosis. A common agreement on what constitutes treatment effect is important. The fact that the criteria for adequate/inadequate pain relief were clearly defined in only eight of 34 trials, and differed for each of the trials, indicates a need for standardisation.

### Psychological factors

No trial specifically addressed psychological variables and the importance of these in the perception of pain. We need to know whether levels of anxiety and/or depression are similar in treatment groups, since this may affect outcome. There is a commonly held belief that the anxiety-reducing and euphoria-producing components of opioid actions account in large part for their analgesic efficacy. This would be interesting to explore in the context of a randomised trial. For example, do psychological factors such as anxiety and depression improve when pain scores improve? Is it possible that patients with specific psychological coping profiles, in particular those who cope anxiously, may have a poor response to opioid therapy? Studies of specific variables such as catastrophic thinking about pain (Sullivan *et al*, 1995) or acceptance of pain (McCracken *et al*, 2004) may be fruitful. The field is recognizing the need to develop assessment techniques that are specific to the context in which the assessment is performed (Mystakidou *et al*, 2005). It is possible to use compound measures that do not have to be lengthy in this setting. In the absence of any multidimensional, psychometrically validated assessment tool, the very minimum requirement would be a unidimensional tool such as a VAS of severity of anxiety or a VAS of severity of depression.

### Other factors influencing opioid treatment outcome

Patients recently or currently receiving radiotherapy and/or chemotherapy were excluded in 20 of 34 trials. Whether patients receiving oncological treatment which may influence pain should be excluded from drug trials on cancer pain depends on the trial design. In studies of long duration, that is, several days or longer, including these patients is a confounding factor. In short studies, for example those examining the effect of short-acting rescue medication for breakthrough pain, including such patients should not be a problem.

A number of other factors, including gender, diurnal variation, pharmacogenetics and opioid pharmacokinetics, may influence the cancer patient's experience of pain and the outcome of opioid therapy. While it is not possible to control for all these variables, some simple measures are available and useful, such as matching groups for gender and controlling plasma opioid concentration at steady state.

### Trial funding

The majority of studies were funded by the pharmaceutical industry. This may represent a source of bias, since research questions of interest for the industry, for example comparing two formulations of the same opioid, may not necessarily coincide with questions of importance for the clinician.

## CONCLUSIONS AND RECOMMENDATIONS FOR FUTURE RESEARCH

Pain is a subjective experience that is affected by many different factors. This makes pain difficult to measure and clinical pain research a challenge. The challenge is even greater in a palliative-care setting where there are special standards of care to maintain, and numerous potential confounding factors.

The data support the clinical experience that it is difficult to perform high-quality scientific trials in palliative-care pain patients. However, it is important to maintain scientific rigour and to ensure that research questions that are relevant to clinical practice are asked.

A number of methodological problems have been identified, including low trial sensitivity, too small trial size and lack of standardised measures of efficacy. Placebo-controlled efficacy trials of oral opioids for cancer pain are lacking. A placebo control is feasible in selected trials. It is important to know which type of pain is being treated and there should be a common definition of opioid efficacy. Psychological factors can influence the experience of pain and should be assessed and reported. A number of other factors have the potential for influencing opioid response, and future research should involve identifying and controlling for such factors.

Having analysed the literature we conclude that there is a need for standardisation and uniformity of design and reporting of trials. Trials must be designed to produce reliable results. This cannot be accomplished by a single researcher, but requires the collaboration of experts in several fields.

### The standard opioid trial design

We propose a consensus meeting where pain researchers, systematic reviewers in pain relief, palliative-care physicians, oncologists, epidemiologists/statisticians and pain psychologists are represented. The objective of such a meeting would be to produce a standard trial design, or set of trials, for opioids in cancer pain. In addition, a checklist for the performance of trials, based on tailor-made validity scores for cancer pain (Antczak *et al*, 1986a, 1986b). The document produced could then be submitted to specialist organizations which have a focus on trial methodology, for example the International Association for the Study of Pain (IASP) and the European Association for Palliative Care (EAPC), and subject to approval, made available on the respective websites. The development and dissemination of a standardised trial, together with checklist for trial performance, will help researchers to plan trials, improve study quality and validity and enable the combination of data from separate trials.

## Figures and Tables

**Table 1:** Characteristics of included trials

	**Trial**	**No. patients enrolled**	**Design**	**Duration**	**Dropouts/ nonevaluable (efficacy)**	**Pain measures**	**Quality score (1–5)**	**Validity score (1–16)**	**Psychological factors; sleep: assessed/reported**
1	Boureau *et al* (1992)	52 (M)	Crossover	14 days	8 (15%)	VASpi; VRSpi	4	13	Mood, four-point scale; ‘Quality and length of sleep’/mean group scores reported: ND
2	Broomhead *et al* (1997)	172 (M)	Parallel group. Placebo control (first phase)	Max. 22 days	20 (12%)	VASpi; VRSpi VRS pain control	4	15	‘Quality of sleep’/Final day group values: ND
3	Bruera *et al* (1996)	95 (M)	Crossover	11 days	21 (22%)	VASpi; VRSpi	4	10	ESSCP/NR
4	Bruera *et al* (1998)	32 (S)	Crossover	14 days	9 (28%)	VASpi; VRSpi	4	11	ESSCP; ‘poor sleep’/NR
5	Bruera *et al* (2004)	103 (M)	Parallel group	4 weeks	37 (36%)	NRSpi	5	15	ESSCP; Folstein Mini-Mental status/ No. of patients having baseline Mini Mental score ⩾27reported. Pain data NR
6	Coluzzi *et al* (2001)	134 (M)	Crossover	Max. 28 days	59 (44%)	NRSpi; VRSpr	5	13	
7	Cundiff *et al* (1989)	23 (S)	Crossover	>4 days	9 (39%)	Nurse rated VRSpi	4	11	
8	Deschamps *et al* (1992)	20 (M)	Crossover	24 days	8 (40%)	VASpi; PPI	5	7	
9	Finn *et al* (1993)	34 (M)	Crossover, placebo control	6 days	3 (9%)	VASpi	5	13	VAS anxiety; VAS depression/ Mean VAS scores±s.e.m.: ND
10	Gabrail *et al* (2004)	47 (M)	Crossover	Max. 30 days	7 (15%)	NRSpi	4	15	BPI scores including pain interference with mood and sleep/Mean (s.d.): ND
11	Gillette *et al* (1997)	35 (M)	Crossover	Max. 17 days	15 (43%)	VASpi; VRSpi	4	8	‘Quality of sleep’ and mean duration of sleep/ND
12	Hagen and Babul (1997)	31 (S)	Crossover	14 days	13 (42%)	VASpi; VRSpi	4	12	ESSCP/NR
13	Hanks *et al* (1987)	27 (S)	Crossover	4 days	9 (33%)	VASpi; VRSpi	4	11	VAS mood; VAS sleep/mean group VAS scores (s.e.): ND
14	Hanks *et al* (1995)	25 (M)	Crossover	6 days	5 (20%)	VASpi	4	8	
15	Hays *et al* (1994)	48 (S)	Crossover	14 days	3 (6%)	VASpi; PPI	4	13	ESSCP/NR
16	Heiskanen and Kalso (1997)	45 (S)	Crossover	Max. 33 days	18 (40%)	VASpi; VRSpi	5	9	MSDEQ/‘one patient reported depression, one reported ‘hollow feeling’’
17	Hoskin *et al* (1989)	19 (S)	Parallel group	12 hours	1 (5%)	VASpi; VRSpi VASpr	4	8	
18	Kalso and Vainio (1990)	20 (S)	Crossover	8 days	1 (5%)	VASpi	3	6	‘Quality of sleep’/less sleep disturbance during oral opioid compared to IV PCA treatment
19	Kaplan *et al* (1998)	180 (M)	Parallel group	6 days	16 (9%)	VRSpi	4	11	0/spontaneous report of nervousness, anxiety
20	Klepstad *et al* (2003)	40 (S)	Parallel group	⩽7 days	6 (15%)	VASpi; VRSpi	5	11	‘Loss of sleep’; HRQOL/HRQOL: ND; sleep data NR
21	Knudsen *et al* (1985)	18 (S)	Crossover	14 days	2 (11%)	VASpi	4	10	
22	Lauretti *et al* (2003)	26 (S)	Crossover	⩾35 days	4 (11%)	VASpi	4	10	
23	Melzack *et al* (1979)	44 (S)	Crossover	‘About’ 4 weeks	14 (32%)	PPI	4	9	
24	Mignault *et al* (1995)	19 (S)	Crossover	10 days	8 (42%)	VASpi; VASpr	4	7	
25	Moriarty *et al* (1999)	100 (M)	Crossover	Max. 9 days	11 (11%)	VASpi; VRSpi	5	15	
26	Mucci-LoRusso *et al* (1998)	101 (M)	Parallel group	Max. 12 days	20 (20%)	VRSpi	5	14	QOL (FACT G)/ND
27	O’Brien *et al* (1997)	85 (M)^*^	Crossover	14 days	16 (19%)	NRSpi	4	12	
28	Parris *et al* (1998)	111 (M)	Parallel group	5 days	37 (33%)	VRSpi	4	16	
29	Portenoy *et al* (1989)	51 (S)	Parallel group	Max. 5 days	2 (4%)	VRSpi	4	11	‘Quality of sleep’/ND
30	Stambaugh *et al* (2001)	30 (S)	Crossover	Max. 35 days	10 (33%)	VASpi; VASr	4	13	
31	Thirlwell *et al* (1989)	23 (M)	Crossover	⩾10 days	5 (22%)	PPI	4	8	
32	Walsh (1985)	36 (S)	Crossover, placebo control	10 days	6 (17%)	VASpi	4	10	VAS mood/anxiety/ND
33	Walsh *et al* (1992)	33 (M)	Crossover	6 days	6 (18%)	VASpi	5	9	VAS anxiety, VAS depression/mean VAS scores: ND
34	Wilder-Smith *et al* (1994)	25 (S)	Crossover	8 days	5 (20%)	VRSpi	4	9	

(S)=single centre study; (M)=multicentre study; pi=pain intensity; pr=pain relief; PPI=present pain intensity (McGill); ^*^not described as multicentre trial, but patients ‘recruited from 30 sites’; BPI=Brief Pain Inventory; ESSCP=Edmonton Staging System for Cancer Pain; ND=no difference between groups; NR=not reported; VAS=visual analogue scale; VRS=verbal rating scale; NRS=numerical rating scale.

**Table 2:** Statistics

	**Trial**	**Design**	**Primary outcome**	**Sample-size calculations**	**Type of statistical analyses**	**Comment on statistical analyses**	**General comments**
1	Boureau *et al* (1992)	CR morphine suspension *vs* CR morphine tablets (E)	Pain intensity	No sample-size calculations mentioned	*t*-test, *χ*^2^ test, ANOVA	Tests appear appropriate	Authors conclude that CRM suspension is as effective as CRM tablets. The tests performed show only no evidence of effect, not evidence of no effect
2	Broomhead *et al*, 1997	SR morphine once a day formulation *vs* SR morphine twice daily formulation (E)	Elapsed time to remedication/total amount of rescue medication (mg)	Sample-size calculations performed, based on results from phase one	ANOVA, Dunnett's multiple comparison procedure, *χ*^2^ test, Fisher's exact test, Cochran-Mantel–Haenzel chi squared	The adjustment of the significance level due to large number of comparisons is appropriate, as are the statistical analyses	
3	Bruera *et al* (1996)	SR hydromorphone *vs* IR hydromorphone (E)	Pain intensity	No sample-size calculations are mentioned	ANOVA, Cochran-Mantel–Haenzel test	Tests appear appropriate	
4	Bruera *et al* (1998)	CR oxycodone *vs* CR morphine (E)	Pain intensity	Appropriate *pre-hoc* calculations appear to have been performed	Three-way-ANOVA, two-way ANOVA, *χ*^2^ test, Pearson correlation	Tests appear appropriate	Authors conclude that the efficacy of CR oxycodone is at least equal to CR morphine. The tests performed show only no evidence of effect, not evidence of no effect
5	Bruera *et al* (2004)	Methadone *vs* morphine (E)	Pain intensity	Appropriate *pre-hoc* calculations appear to have been performed	*χ*^2^ test, Pearsons rho, Wilcoxon rank-sum test, Fisher's exact test	Tests appear appropriate	
6	Coluzzi *et al*, 2001	OTFC *vs* IR morphine for breakthrough pain (E)	Pain intensity	Appropriate *pre-hoc* calculations appear to have been performed	Three-way-ANOVA	Tests appear appropriate	
7	Cundiff *et al* (1989)	CR morphine *vs* IR morphine (E)	Pain intensity	No sample-size calculations mentioned	Two-way ANOVA, parallel line log-ratio assay (some kind of ANOVA)	Old reference (Finney), difficult to distinguish the method from other ANOVA	
8	Deschamps *et al*, 1992	IR release *vs* CR release morphine (E)	Pain intensity	No sample-size calculations mentioned	Repeated-measures ANOVA, paired *t*-test	Tests appear appropriate	
9	Finn *et al* (1993)	SR morphine tablets compared with IR morphine solution (E)	Pain intensity	No sample-size calculations mentioned	Linear regression, McNemars test, ANOVA	Tests appear appropriate	
10	Gabrail *et al* (2004)	ER oxymorphone *vs* CR oxycodone (E)	BPI (pain intensity and interference)	No sample-size calculations mentioned	Mixed-effects model	The authors ignore a trend because it is stated to be ‘not clinically significant’. This is not supported by analyses	Authors conclude that oxymorphone ER and oxycodone CR were considered equivalent if the confidence interval around the treatment difference included zero. This kind of two-sided test can only tell whether the two are different, not whether they are equivalent
11	Gillette *et al* (1997)	Oral morphine syrup *vs* SR morphine capsules (E)	Pain intensity	No sample-size calculations mentioned	Linear regression, Spearman's rank order correlation test	A ‘test’ for bioequivalence is mentioned, however not justified. Other tests appear appropriate	
12	Hagen and Babul (1997)	CR oxycodone *vs* CR hydromorphone (E)	Pain intensity	Appropriate *pre-hoc* calculations appear to have been performed l	Three-way-ANOVA, two-way ANOVA, Fisher's exact test, *χ*^2^ test, binomial test	Tests appear appropriate	
13	Hanks *et al* (1987)	CR morphine *vs* IR morphine solution (E)	Pain intensity	No sample-size calculations mentioned	Mann–Whitney *U*-test, ‘standard crossover-design nonparametric techniques’ (two-sample *t*-test)	Six different outcomes were tested, no adjustments were performed	
14	Hanks *et al* (1995)	SR morphine tablet (200 mg) *vs* two 100 mg tablets (E)	Pain intensity	No sample-size calculations mentioned	ANOVA, Wilcoxon signed-rank test, trapezoidal method for AUC, *t*-test	The statistical analyses seem appropriate	
15	Hays *et al* (1994)	CR hydro-morphone *vs* IR hydro-morphone (E)	Pain intensity	Appropriate *pre-hoc* calculations appear to have been performed	Three-way ANOVA	Tests appear appropriate	Authors conclude that CR hydromorphone is as effective as IR hydromorphone. The tests performed show only no evidence of effect, not evidence of no effect
16	Heiskanen and Kalso (1997)	CR oxycodone *vs* CR morphine (E)	Pain intensity	No sample-size calculations mentioned	Mann–Whitney *U*-test, Wilcoxon signed-rank test, paired *t*-test, *χ*^2^ test, regression analysis, one-way and two-way crossover ANOVA	Tests appear appropriate. Not possible to ascertain which tests used at what time	
17	Hoskin *et al*, 1989	CR morphine+IR morphine *vs* CR morphine+placebo	Pain intensity	No sample-size calculations mentioned	Trapezoidal method for AUC, regression (least squares), *t*-test	Tests appear appropriate	
18	Kalso and Vainio (1990)	Morphine *vs* oxycodone (E)	Pain intensity	No sample-size calculations mentioned	Wilcoxon signed-rank test, rank-sum test, *t*-test, Spearmans rank correlations, linear regression	Tests appear appropriate	
19	Kaplan *et al* (1998)	CR oxycodone *vs* IR oxycodone (E)	Pain intensity	No sample-size calculations mentioned	ANOVA (two-way, repeated measures), Fisher's exact test, Kruskal–Wallis test	Tests appear appropriate	Authors conclude that CR oxycodone is as effective as IR oxycodone. The tests performed show only no evidence of effect, not evidence of no effect
20	Klepstad *et al* (2003)	SR morphine *vs* IR morphine (E)	Time needed to achieve pain relief	Appropriate *pre-hoc* calculations appear to have been performed	*t*-test, Mann–Whitney *U*-test	Tests appear appropriate	Authors conclude that SRM given daily and IRM given 4-hourly are equally effective. The tests performed show only no evidence of effect, not evidence of no effect
21	Knudsen *et al* (1985)	SR morphine tablets *vs* IR morphine tablets or suspension (E)	Pain intensity	No sample-size calculations mentioned	Wilcoxon paired rank-sum test	Tests appear appropriate	Difficult to understand why the significant finding is not clinically meaningful
22	Lauretti *et al* (2003)	SR morphine *vs* SR oxycodone (E)	Consumption of rescue medication	No sample-size calculations mentioned	Mann–Whtiney *U*-test, Wilcoxon signed-rank test, *χ*^2^ test	Adverse effects analysed with *χ*^2^ test, this is not appropriate on small samples	
23	Melzack *et al* (1979)	Brompton mixture *vs* morphine (E)	Pain intensity	Stated that a subject group of 20 is substantial in a crossover design. No sample-size calculations mentioned	*t*-test, *χ*^2^ test	Tests appear appropriate	No reason stated for choosing *P*-value of 0.01, however, several outcomes were tested, therefore appropriate to use a lower level
24	Mignault *et al* (1995)	SR morphine (MSC) 8-hourly *vs* 12-hourly administration (E)	Pain intensity	Some posterior power calculations performed	Pairwise *t*-test, McNemars test	Tests appear appropriate	
25	Moriarty *et al* (1999)	CR hydromorphone *vs* CR morphine (E)	Pain intensity	Appropriate *pre-hoc* calculations appear to have been performed	Koch nonparametric method for crossover studies, binomial test	Tests appear appropriate	Authors conclude that hydromorphone and morphine are equally effective. The tests performed show only no evidence of effect, not evidence of no effect
26	Mucci-LoRusso *et al* (1998)	CR oxycodone *vs* CR morphine (E)	Pain intensity	Appropriate *pre-hoc* calculations appear to have been performed	Two-way ANOVA, Kaplan–Meier and Logrank-test, Fishers exact test, linear regression	Tests appear appropriate	Authors mention Kaplan–Meier estimate and log-rank test under ‘statistical analysis’. Results of these analyses unclear
27	O’Brien *et al* (1997)	MXL morphine dosed once daily *vs* MST continuous dosed twice daily (E)	Use of escape medication	No sample-size calculations mentioned	Double triangular sequential test, Koch method for crossover studies, McNemars test, *χ*^2^ test, binomial test	Stated that the study should have stopped after 33 patients. However, continued until 69 patients. This may be against protocol	
28	Parris *et al* (1998)	CR oxycodone *vs* IR oxycodone (E)	Pain intensity	Appropriate *pre-hoc* calculations appear to have been performed	Fisher's exact test, two-way ANOVA, two-way ANCOVA	Tests appear appropriate	
29	Portenoy *et al* (1989)	SR morphine tablet (100 mg) *vs* three 30 mg tablets (E)	Pain intensity	Some posterior power calculations performed	*t*-test, *χ*^2^ test, repeated-measures ANOVA, Fisher's exact test, Wilcoxon rank sum test	Tests appear appropriate	
30	Stambaugh *et al* (2001)	CR oxycodone every 12 h *vs* IR oxycodone given qid (E)	Pain intensity	A comment on sample size was presented, however, it remains unclear whether any calculations were performed	ANOVA, signed rank test	Tests appear appropriate	
31	Thirlwell *et al* (1989)	Oral morphine solution *vs* CR morphine tablets (E)	Pain intensity	No sample-size calculations mentioned	Trapezoidal method for AUC, *t*-test, repeated-measures ANOVA, Wilcoxon signed-rank test, linear regression	Tests appear appropriate. The adjustment of the significance level due to large number of comparisons is appropriate	
32	Walsh (1985)	Oral aqueous solution of morphine compared to SR morphine tablets (E)	Pain intensity	No sample-size calculations mentioned	Paired and unpaired *t*-test	Results of analyses not presented	
33	Walsh *et al* (1992)	SR morphine dosed every 12 h *vs* IR morphine dosed 4 hourly (E)	Pain intensity	Some posterior power calculations performed	ANOVA, *χ*^2^ test, McNemars test	Tests appear appropriate	Authors conclude that SRMS is as effective as IRMS. The tests performed show only no evidence of effect, not evidence of no effect
34	Wilder-Smith *et al* (1994)	Tramadol *vs* morphine (E)	Pain intensity	No sample-size calculations mentioned	Wilcoxon signed-rank test	The adjustment of the significance level due to large number of comparisons is appropriate, as are the statistical analyses	

(E)=equivalency study; (ER)=extended release; (CR)=controlled release; (IR)=immediate release; (SR)=slow –release; BPI=Brief Pain Inventory; ANOVA=analysis of variance; AUC=area under the curve; OTFC=oral transmucosal fentanyl citrate; CRM=controlled-release morphine.
